# Endemic mycoses in South Africa, 2010–2020: A decade-long description of laboratory-diagnosed cases and prospects for the future

**DOI:** 10.1371/journal.pntd.0010737

**Published:** 2022-09-28

**Authors:** Rutendo E. Mapengo, Tsidiso G. Maphanga, Wayne Grayson, Nelesh P. Govender

**Affiliations:** 1 National Institute for Communicable Diseases (Centre for Healthcare-Associated Infections. Antimicrobial Resistance and Mycoses), a Division of the National Health Laboratory Service, Johannesburg, South Africa; 2 School of Pathology, Faculty of Health Sciences, University of the Witwatersrand, Johannesburg, South Africa; 3 Ampath National Reference Laboratory, Pretoria, South Africa; 4 Division of Medical Microbiology, Faculty of Health Sciences, University of Cape Town, Cape Town, South Africa; 5 Medical Research Council Centre for Medical Mycology, University of Exeter, Exeter, United Kingdom; 6 Institute of Immunity and Infection, St George’s University of London, London, United Kingdom; International Foundation for Dermatology, London, United Kingdom, UNITED KINGDOM

## Abstract

**Background:**

Emergomycosis, histoplasmosis, sporotrichosis and blastomycosis are endemic to southern Africa; the first two are AIDS-related mycoses. We described laboratory-diagnosed cases of endemic and imported mycoses in South Africa over a decade and discuss available diagnostic tools, reasons for the current under-estimation of cases and future strategies to improve case ascertainment.

**Materials and methods:**

We analysed electronic pathology laboratory data from all public laboratories and one large private laboratory in South Africa from 2010–2020. Diagnostic specimens processed at the national mycology reference laboratory were also included. We classified cases as proven, probable and possible based on the method of identification.

**Results:**

We identified 682 cases, of which 307 were proven, 279 were probable and 96 were possible. Of 307 culture-confirmed cases, 168 were identified by phenotypic methods plus sequencing, 128 by phenotypic methods alone and 11 by direct PCR. Of 279 probable cases, 176 had yeasts observed on histology, 100 had a positive *Histoplasma* antigen test and 3 a positive pan-dimorphic PCR test. All 96 possible cases had compatible clinical syndrome with inflammatory infiltrates on skin tissue histology. A majority of cases had an unspecified endemic mycosis (207/682, 30.4%), followed by sporotrichosis (170/682, 24.9%), emergomycosis (154/682, 22.6%), histoplasmosis (133/682, 19.5%), blastomycosis (14/682, 2.1%) and talaromycosis (4/682, 0.6%).

**Conclusions:**

This study reports a relatively low number of cases over a decade considering an estimated large population at risk, suggesting that a substantial fraction of cases may remain undiagnosed. There is a need to increase awareness among healthcare workers and to develop rapid point-of-care diagnostic tools and make these widely accessible.

## Author summary

Emergomycosis, histoplasmosis, sporotrichosis and blastomycosis are serious fungal diseases which occur in southern Africa among people with either healthy or weakened immune systems. These fungal diseases may be missed because they mimic other diseases, occur together with other conditions that mask their presence or are simply not considered by healthcare workers. There are very few available simple diagnostic tests and most African countries do not conduct fungal disease surveillance. We described cases of these fungal diseases, which had been diagnosed in South African laboratories, from 2010–2020. We identified 682 cases, of which 307 were proven, 279 were probable and 96 were possible based on the available clinical and laboratory information. A majority of cases could not be allocated to one of the four disease types and were classified as unspecific endemic mycoses (30.4%). The remainder were sporotrichosis (24.9%), emergomycosis (22.3%), histoplasmosis (19.5%), blastomycosis (2.1%) and a travel-associated infection, talaromycosis (0.6%). We found relatively few cases considering the large population at risk. We believe that this could be due to the above-mentioned issues and insufficient expertise in diagnostic laboratories. There is a need to increase awareness among healthcare workers and to develop rapid point-of-care diagnostic tools and make these widely accessible.

## Introduction

Endemic mycoses are caused by thermally-dimorphic fungi that are generally restricted to certain geographical areas. Over the last few decades, reports of endemic mycoses have increased in Sub-Saharan Africa partly due to gradually increasing awareness of serious fungal diseases among healthcare workers with expanded advocacy efforts and increasing availability of improved diagnostic techniques although these are still not optimal [1–5]. There has been an expansion of areas in which these diseases are recognized to be endemic owing to climate change, migration and travel [6]. Some endemic mycoses such as histoplasmosis disproportionately affect immunocompromised hosts, for example, people living with HIV [1–6]. According to UNAIDS, the global burden of HIV in 2020 was 37.7 million, with 36.0 million adults and 1.8 million children aged <15 years [7]. UNAIDS estimated that South Africa had around 7.5 million individuals living with HIV in 2020, 92% of whom were aware of their HIV status, 70% of whom were on HIV treatment and 64% of whom were virally suppressed [8]. The medical impact and burden of endemic mycoses is unknown or underestimated in many African countries [9]. This is because endemic mycoses may mimic other diseases or occur together with other conditions resulting in few diagnoses, suboptimal patient management and poor clinical outcomes [10].

In southern Africa, emergomycosis, histoplasmosis, sporotrichosis and blastomycosis are endemic [11]. Based on case reports and series, Schwartz *et al* estimated 100, 60, 40 and 10 diagnosed cases per year for emergomycosis, histoplasmosis, sporotrichosis and blastomycosis respectively in South Africa [11]. These diseases are usually diagnosed by culture or histopathology which requires skilled personnel for accurate diagnosis and pathogen identification [12]. Several studies over the past decade have characterized novel dimorphic fungal pathogens causing severe infections among South African patients [1,3,13–16]. The emergence of novel endemic pathogens warrants an intermittent review of the epidemiology to determine changes in disease distribution and patterns [17–20]. Relatively few outbreaks of endemic mycoses have been reported in southern Africa. Sporadic cases of acute pulmonary histoplasmosis were reported mainly among new members of speleological societies visiting caves around the country [21]. An outbreak of lymphocutaneous sporotrichosis among mine workers in South Africa was described in 2015 [22]. Travel-associated infections also occur. For example, a fatal case of talaromycosis was reported in an HIV-seropositive South African patient with a history of recent travel to mainland China [23].

There are few studies on endemic mycoses on the African continent [24,25]. These infections are not included on the list of notifiable medical conditions in South Africa. The National Institute for Communicable Diseases (NICD) initiated passive surveillance for endemic mycoses in 2014, first requesting diagnostic pathology laboratories to send cultured dimorphic isolates for molecular identification and later, clinical specimens from suspected cases for reference diagnostic testing. For the latter purpose, a commercial *Histoplasma* enzyme immunoassay (EIA) and then an in-house pan-dimorphic reverse transcriptase-quantitative (RT-qPCR) assay was introduced for investigational use at NICD in 2012 and 2020 respectively. To understand the epidemiology and characteristics of endemic mycoses in South Africa, we performed a comprehensive review of cases diagnosed at public and private laboratories over a decade.

## Materials and methods

### Ethics statement

We obtained approval for this retrospective record review from the human research ethics committees of the University of Free State (UFS-HSD2018/0992) and the University of the Witwatersrand (M140112 and M210109).

### Study design and setting

We performed a retrospective electronic review of laboratory-diagnosed cases of diseases caused by thermally-dimorphic fungi, which were reported by microbiology and histopathology laboratories affiliated either to the National Health Laboratory Service (NHLS) or one large national private pathology practice in South Africa, from 1 January 2010 through to 31 December 2020. Laboratory data were obtained from the NHLS corporate data warehouse, which archives pathology test results from all public-sector facilities, and from the electronic laboratory information system of the private pathology practice which serves an estimated 40% of the private healthcare market in South Africa. We also included referred cases that had been diagnosed at the NICD. Where available, we extracted data on patient demographics, clinical presentation and HIV infection status from laboratory request forms submitted to NICD or from final histology laboratory reports. In addition, for public-sector cases, we looked for NHLS haematology, histopathology, chemical pathology and microbiology test results performed within two weeks before or after confirmation of the infection. We created an online Google forms survey for NHLS laboratories to understand diagnostic practices for endemic mycoses (S1 Text).

### Case definitions

A case was defined as a patient of any age with any specimen type submitted to a laboratory from which a thermally-dimorphic fungus was cultured or detected by histopathology, *Histoplasma* antigen EIA, pan-dimorphic reverse-transcriptase quantitative PCR (RT-qPCR) and pan-dimorphic fungal PCR/sequencing. We classified cases as: 1) proven if a thermally-dimorphic fungus was cultured (identified by phenotypic or molecular methods) from a clinical specimen or if fungal DNA was detected directly from a specimen by PCR and identified by sequencing of the fungal internal transcribed spacer (ITS) region, large subunit of the ribosomal RNA (LSU) or calmodulin gene [26,27], 2) probable if morphologically-consistent yeasts were observed on histopathological examination or if a urine *Histoplasma* antigen test or a pan-dimorphic (RT-qPCR) assay on several specimen types was positive, and 3) possible if a compatible clinical syndrome was reported with a consistent tissue inflammatory infiltrate but no yeasts were observed on histology. We excluded duplicate cases and non-patient samples distributed to laboratories as part of an external quality assessment programme.

### Culture and fungal identification methods

Fungal culture and identification was performed at diagnostic laboratories according to their standard operating procedures. Only difficult-to-identify isolates were referred to NICD for confirmation by phenotypic or molecular methods. At NICD, isolates were sub-cultured onto potato dextrose agar (Diagnostic Media Products [DMP], Sandringham, South Africa) and incubated at 25°C and 30°C for 4 weeks. To confirm thermal dimorphism, the isolate was inoculated onto brain heart infusion (BHI) agar (DMP) or BHI + 5% sheep blood (DMP) and incubated at 37°C for up to 4 weeks for conversion to the yeast phase. All cultured isolates were processed in a biosafety class II cabinet with use of personal protective equipment including N95 masks, laboratory coats and gloves. The yeast and mycelial features were examined by light microscopy after staining with lactophenol cotton blue (Difco, Becton Dickinson, Franklin Lakes, United States) or a Gram stain (DMP). For molecular identification, DNA was extracted directly from fungal isolates using the Zymo ZR fungal/bacterial DNA miniprep kit (Zymo Research Corp., Irvine, USA) following manufacturer’s instructions. PCR amplification and sequencing was performed either by targeting the *ITS/LSU* genes for all dimorphic fungi and the calmodulin gene for *Sporothrix schenckii* species-complex [26, 27]. The DNA amplicons were sequenced using a 3130 sequencer (Applied Biosystems, Life Technologies Corporation, USA). Sequences were subjected to BLAST analyses in GenBank (www.ncbi.nlm.nih.gov).

### Pan-dimorphic PCR assay

We modified the published protocol for a novel *Histoplasma* reverse transcriptase-quantitative PCR assay to target the mitochondrial small subunit of *Histoplasma* and *Emergomyces* and used this pan-dimorphic assay on cultured isolates and directly on clinical specimens including fresh tissue, formalin-fixed paraffin-embedded (FFPE) tissue, bone marrow aspirates, whole blood or plasma and urine [28,29]. Briefly, DNA from tissue samples was extracted using the Qiagen DNA FFPE tissue kit (Qiagen, Hilden, Germany) as per the manufacturer’s instructions. Nucleic acids were extracted by taking 1.6 ml of liquid samples, a loopful of the cultured isolate or a small piece of FFPE tissue and adding to a 2 ml Eppendorf tube containing beads. The mixture was subjected to bead beating at 3000 rpm for 2 minutes. The samples were centrifuged at 10 000 rpm for 5 minutes. Nucleic acids were then extracted from 200 μl of the sample using a High Pure Viral Nucleic Acid kit (Roche Molecular Systems, Branchburg, United States) according to the manufacturer’s instructions. Nucleic acids were amplified using the specific *Histoplasma*/*Emergomyces* primers and probe in the LightCycler 480 Instrument II (Roche Molecular Systems). Any amplification with a Cq value of less than 40 was considered positive for *Histoplasma/Emergomyces*. Since the validation results for this in-house assay have not yet been published, patients with positive PCR results were considered as meeting the probable case definition.

### *Histoplasma* antigen enzyme immunoassay

Urine specimens from cases with suspected histoplasmosis were submitted to the NICD for *Histoplasma* antigen testing between 2012 and 2020. Urine specimens collected from 2012 to July 2014 were tested using the Alpha *Histoplasm*a antigen assay (IMMY, Norman, United States) and those collected from August 2014 to December 2020 were tested with the Clarus *Histoplasma* antigen assay (IMMY). Urine specimens were batched and processed weekly according to manufacturer’s instructions and as described by Maphanga et al., 2020 [30]. Since both Alpha and Clarus assays cross react in urine with dimorphic fungi such as *Emergomyces*, *Blastomyces*, *Sporothrix* and *Talaromyces marneffei*, patients with positive EIA results were considered as meeting the probable case definition [31].

### Statistical analysis

Descriptive statistics were used. Categorical variables were reported as frequencies and proportions, while non-normally distributed quantitative variables were presented as medians and interquartile ranges (IQR). Statistical analyses were conducted using R (R studio, Boston, United States) and STATA (StataCorp, College Station, United States).

## Results

### Patient demographic and clinical characteristics

**Fig 1:**
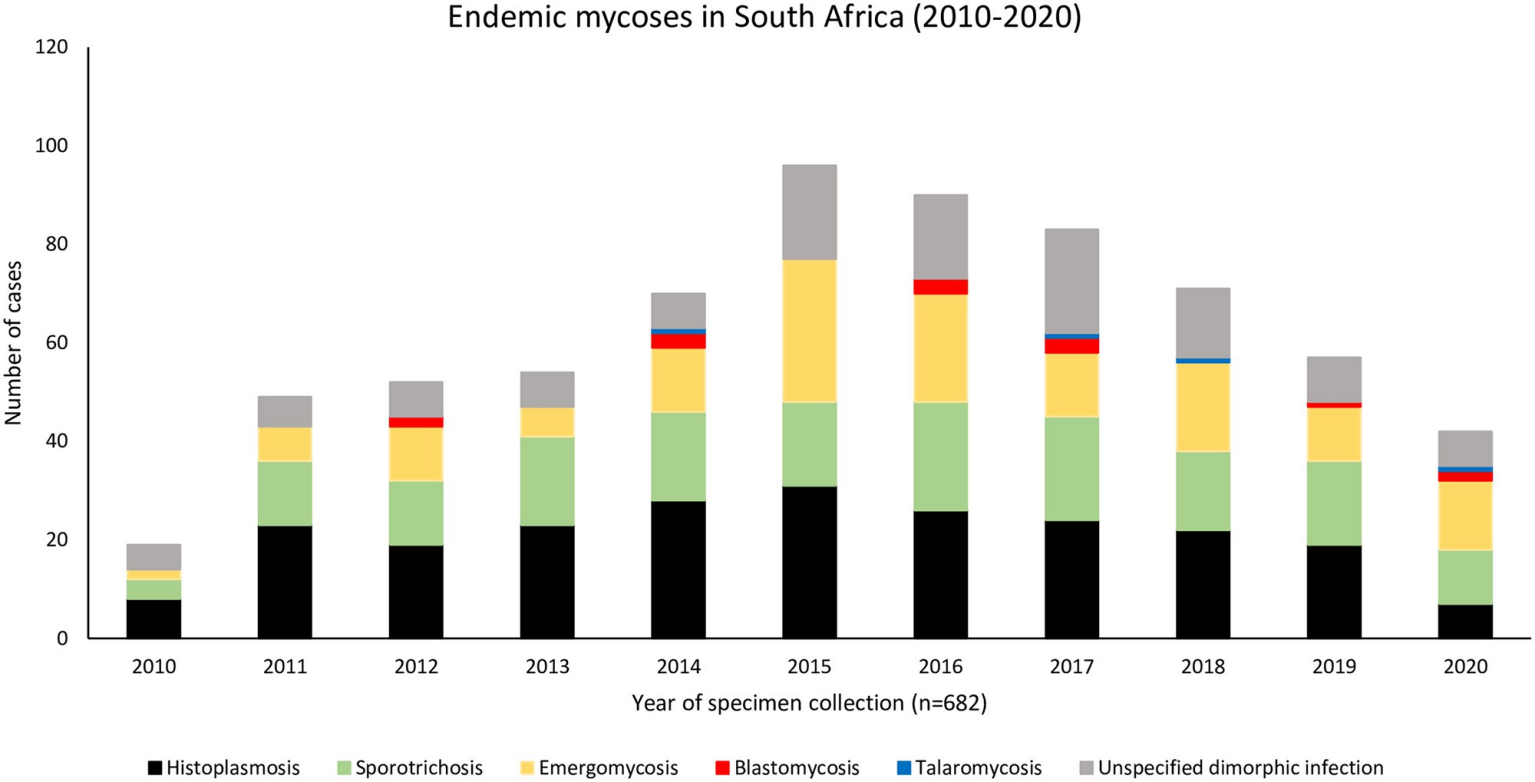
Frequency of laboratory-diagnosed endemic mycoses (proven, probable and possible) per year in South Africa from 2010–2020, n = 682.

**Fig 2:**
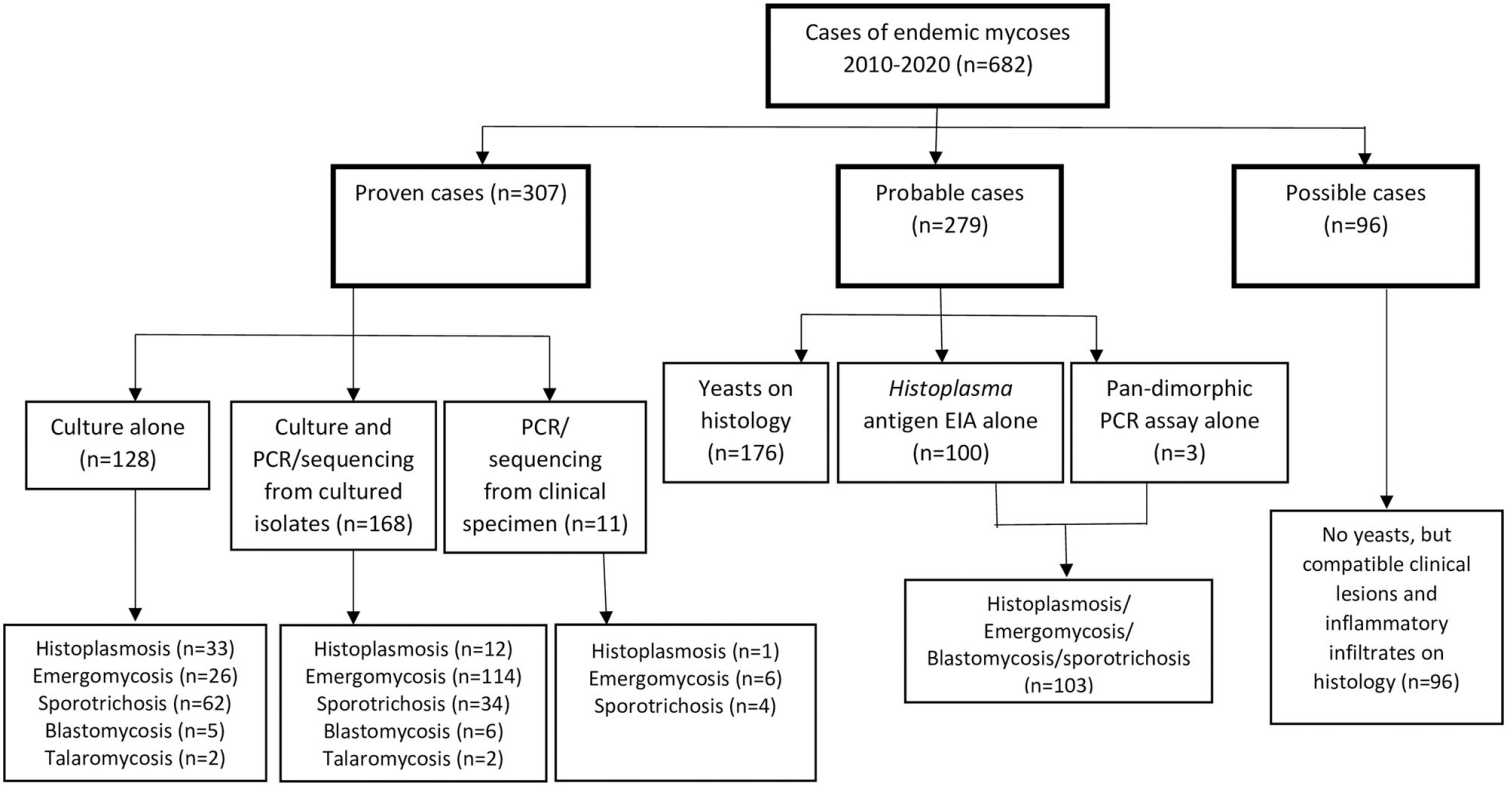
Flow chart showing levels of certainty for cases of endemic mycoses reported in South Africa from 2010–2020, n = 682.

Between January 2010 and December 2020, we identified 682 cases of endemic mycoses (Fig 1). Overall, the patients had a median age of 37 years (IQR, 31–45), and 436 (64%) were male. Three hundred and seven (45%) cases were classified as proven, 279 (41%) as probable and 96 (14%) as possible (Fig 2). Eighty-six per cent (586/682) of cases were diagnosed at public hospitals. A majority were from Gauteng Province (247/682, 36%) followed by Western Cape (154/682, 23%), KwaZulu-Natal (125/682, 18%), Eastern Cape (85/682, 12%), Free State (19/682, 3%), Mpumalanga (17/682, 2%), North West (13/682, 2%), Limpopo (11/682, 2%) and Northern Cape provinces (6/682, 1%) (S1 Fig.). Specimen types were available for 569 (83%) of the 682 cases. The most commonly-submitted specimens were skin tissue (276/573, 48%) followed by unspecified tissue (103/573, 18%), urine (102/573, 18%) and blood (55/573, 10%). Most cases were diagnosed by histopathology alone (273/682, 40%), followed by culture plus phenotypic and panfungal PCR/sequencing identification (167/682, 24%), culture plus phenotypic identification alone (128/682, 18%), *Histoplasma* antigen tests (100/682, 15%) and a pan-dimorphic RT-qPCR test (3/682, 0.4%) (Fig 2). The detailed clinical characteristics and demographics of all patients are summarised in Table 1.

**Table 1:** Demographic and clinical characteristics of patients with proven, probable or possible laboratory-diagnosed endemic mycoses in South Africa (2010–2020), n = 682.

Characteristics	All N = 682	Unspecified endemic mycoses n = 207	Sporotrichosis n = 170	Emergomycosis n = 154	Histoplasmosis n = 133	Blastomycosis n = 14	Talaromycosis n = 4
**Age, n**	643	202	161	137	126	13	4
**Age in years; median [IQR]**	37 (31–45)	36 (30–44)	45 (34–57)	36 (30–41)	36 (31–41)	34 (29–51)	36 (26–40)
**Missing data, n**	39	5	9	17	7	1	-
**Sex, n**	670	204	170	147	131	14	4
**Female, n (%)**	234 (35)	74 (36)	46 (27)	48 (33)	60 (46)	4 (29)	2 (50)
**Male, n (%)**	436 (64)	130 (64)	124 (73)	99 (67)	71 (54)	10 (71)	2 (50)
**Missing, n**	12	3	-	7	2	-	-
**Health sector**	682	207	170	154	133	14	4
**Public, n (%)**	586 (86)	193 (93)	107 (63)	143 (93)	128 (96)	12 (86)	3 (75)
**Private, n (%)**	96 (14)	14 (7)	63 (37)	11 (7)	5 (4)	2 (14)	1 (25)
**HIV status**	377 (55)	125	48	122	72	7	3
**Seropositive, n (%)**	368 (98)	122 (98)	47 (98)	122 (100)	72 (100)	2 (29)	3 (100)
**Seronegative, n (%)**	9 (2)	3 (2)	1 (2)	-	-	5 (71)	-
**Missing, n**	305	82	122	32	-	7	1
**Median CD4 cell** ^a^	181	23	38	100	17	2	1
**Median CD4 count at diagnosis in cells/mm**^**3**^ **(IQR)**	24 (9–66)	23 (5–67)	59 (18–98)	14 (7–40)	50 (8–98)	78 (67–89)	5 (5–5)
**Missing, n**	501	184	132	54	116	12	3
**ART** ^a^	85	-	19	56	8	2	
**Naive, n (%)**	14 (16)	-	3 (16)	9 (16)	1 (13)	1 (50)	-
**Experienced, n (%)**	71 (84)	-	16 (84)	47 (84)	7 (88)	1 (50)	-
**Missing, n**	597	207	151	98	125	12	4
**Specimen type**	569	188	154	140	72	11	4
**Skin**	276 (49)	75 (39)	86 (56)	54 (39)	52 (72)	9 (82)	-
**Tissue**	103 (18)	17 (9)	41 (27)	28 (20)	15 (21)		-
**Urine**	100 (18)	89 (47)	3 (2)	8 (6)	-	-	-
**Blood**	55 (10)	-	9 (6)	42 (30)	1 (0.6)	-	3 (75)
**Bone marrow**	12 (2)	5 (3)	1 (0.7)	4 (3)	2 (3)	-	1 (25)
**Bone fragments**	2 (0.4)	1 (0.5)	1 (0.7)	-	-	-	-
**Pus abscess**	9 (2)	-	7 (5)	-	1 (1.4)	-	1 (25)
**Nails** ^b^	5 (0.9)	-	1 (0.7)	3 (2)	-	1 (9)	-
**Sputum**	3 (0.5)	1 (0.5)	1 (0.7)	1 (0.7)	-	-	-
**Wound swabs**	3 (0.5)	-	3 (2)	-	-	-	-
**Cerebrospinal fluid**	1 (0.2)	-	1 (0.6)	-	-	-	-
**Hair** ^c^	1 (0.2)	-	-	-	1 (0.6)	-	-
**Missing**	113	19	16	14	61	3	-
**Outcome, n**	57	-	4	47	1	4	1
**Died, n (%)**	30 (61)	-	4 (100)	24(51)	1 (100)	3 (75)	1 (100)
**Survived, n (%)**	19 (39)	-	-	23 (49)	-	1 (25)	-
**Missing, n**	633 (93)	207	166	107	132	10	3

^a^: ART status and CD4 counts only for HIV-seropositive patients on presentation;

^b and c^: diagnostic laboratories reported positive culture of a thermally-dimorphic fungus from nail or hair specimens; the fungal identification was not confirmed at a reference laboratory and thus these may not truly be cases.

### Sporotrichosis

Sporotrichosis was the most commonly-diagnosed endemic mycosis occurring in 25% (170/682) of all cases. Of these, 73% (124/170) of the individuals were male, with a male to female ratio of 3:1 and an overall median age of 45 years (IQR, 34–57). Gauteng and KwaZulu-Natal provinces had the highest number of sporotrichosis cases with 38% (65/170) and 28% (48/170), respectively (S1 Fig). Fifty-nine per cent (101/170) of these cases were proven as *S*. *schenckii* by culture plus phenotypic and panfungal PCR/sequencing methods. Thirty-nine (23%) cases were categorised as probable and 29 (17%) as possible cases. The most common specimen types submitted for diagnosis of sporotrichosis were skin tissue (77/170, 45%) and unspecified tissue (41/170, 24%). HIV infection status was available for 28% (48/170) of the patients and of these, 98% (47/48) were HIV-seropositive. Forty-five of the HIV-seropositive cases were proven cases of sporotrichosis, 24 of these at NICD. Of the 47, 38 (81%) had a recorded CD4 count with a median of 59 cells/μl (IQR, 18–98 cells/μl). Forty per cent (19/47) of the HIV-seropositive cases had known ART status and of these, 84% (16/19) were ART-experienced. Seventy-eight per cent (25/35) of the patients with available data had multiple skin lesions, which were mostly located on the face and upper and lower limbs. Serum albumin levels were available for ten patients (6%) and all were below the specified reference ranges. Forty-four and forty-two patients had serum alkaline phosphatase and γ-glutamyl transferase results respectively and all had elevated levels for both.

### Emergomycosis

Twenty-three per cent (154/682) of all patients in this case series had emergomycosis. Ninety-four per cent were proven cases (144/154) and 6% (10/154) were probable cases. Eighty-two per cent (119/154) of proven cases had species-level identification established using culture and phenotypic methods plus a panfungal PCR/sequencing assay. Of these, 98% (117/119) were infected with *Emergomyces africanus* and 2% (2/119) had *Emergomyces pasteurianus*. The male to female ratio was 2:1 (99:48). Diagnoses were mainly made at public hospitals (93%, 143/154). Patients had a median age of 36 years (IQR, 30–41) and were mostly diagnosed in the Western Cape Province (75/154, 49%), followed by Gauteng Province (45/154, 29%) (S1 Fig). Blood cultures (42, 29%), skin tissue (40, 29%) and unspecified tissue (28, 20%) were the main specimen types for diagnosis of emergomycosis. Of the 154 patients, 79% (122/154) had a known HIV infection status and all were HIV-seropositive. Forty-six per cent (56/122) of the HIV-seropositive cases had their ART status recorded. Of these 56 cases, 47 (84%) were ART-experienced and nine patients (16%) were ART-naive. Eighty-two per cent (100/122) of the cases had a recorded CD4 count at the time of diagnosis and the median CD4 count was 14 cells/μl (IQR, 7–40 cells/μl). Eighty-eight per cent (108/122) had multiple skin lesions, 6% (6/108) had recorded pulmonary disease and 27% (29/108) had both cutaneous and pulmonary disease. Five per cent (5/108) of the patients had fungaemia. One patient with culture-confirmed *E*. *africanus* fungaemia had a history of a chronic headache, and brainstem lesions with no ring enhancement were visualised on a computer tomography scan with contrast. The remaining 22% (24/108) had no or limited available clinical information. Thirteen of these 24 patients had non-specific symptoms which included fever, weakness, weight loss, cough, nausea, diarrhoea and night sweats. Serum alkaline phosphatase results were available for 47/154 (30%) patients and the median value was high at 163 units per litre (IQR, 121–287). High levels of serum γ-glutamyl transferase were also reported in 48/154 (31%) with a median of 145 units per litre (IQR, 102–281). Clinical outcomes were available for 39 patients and of these, 54% (21/39) died.

### Histoplasmosis

There were 133 (20%) cases of histoplasmosis during the study period. Patients had a median age of 36 years (IQR, 31–41) and a majority were male (71/133, 53%). Ninety-six per cent of cases were diagnosed at public hospitals. Although histoplasmosis was diagnosed in all nine provinces, most cases were reported from KwaZulu-Natal (39/133, 29%) and Western Cape provinces (32/133, 24%) (S1 Fig). Of the 133 cases, 46 (35%) were proven, 67 (50%) were probable and 20 (15%) were possible. Proven cases included a majority (72%; 33/46) identified by culture plus phenotypic identification methods alone, 26% (12/46) identified by culture plus phenotypic and panfungal PCR/sequencing methods and a single case confirmed by panfungal PCR/sequencing alone (directly from tissue). These proven cases were all caused by *Histoplasma capsulatum*. Among probable cases, all were diagnosed by histology and yeasts were observed with a morphology most consistent with *Histoplasma* species. Of the 133 patients with histoplasmosis, 64% (72/133) had a known HIV status and all 72 were HIV-seropositive. Only 17 cases had a known CD4 count at the time of diagnosis with a median CD4 count of 50 cells/μl (IQR, 8–98 cells/μl). Antiretroviral treatment status was available for 11% (8/72) and 88% (7/8) of patients were ART experienced. Of the 133 cases, clinical symptoms at presentation were recorded for only 6% (8/142). All eight patients had widespread skin lesions, while two were also reported to have TB. Twenty-two patients had full blood count results available and all patients had evidence of pancytopenia. Overall, the blood chemistry and liver function test results were available for 17% (22/133) patients. Eight per cent (11/133) of the patients had results for serum levels of conjugated bilirubin and all were elevated; 14% (18/133) patients with results for serum alkaline phosphatase also had elevated levels (Table 2).

**Table 2:** Laboratory tests performed 2 weeks before or after the fungal diagnostic test for patients with proven, probable or possible laboratory-diagnosed endemic mycoses in South Africa, 2010–2020.

Characteristics	All	Emergomycosis	Sporotrichosis	Histoplasmosis	Blastomycosis	Talaromycosis
**Blood chemistry & liver function tests**						
**Creatinine— μmol/litre (n)**	130	57	46	19	4	3
Reference range	49–90					
Median	76	89	72	65	60	75
Interquartile range	53–111	61–128	52–105	45–87	53–1023	70–156
**Albumin— μmol/litre (n)**	36	16	10	5	4	1
Reference range	35–52					
Median	**26**	**24**	**31**	**26**	**26**	**14**
Interquartile range	19–31	18–28	26–39	18–29	21–35	14–14
**Total bilirubin— μmol/litre(n)**	114	50	42	17	3	2
Reference range	0–21					
Median	7	8	6	11	7	5
Interquartile range	5–13	5–16	4–10	7–14	3–31	4–5
**Conjugated bilirubin— μmol/litre (n)**	99	45	38	11	3	2
Reference range	0–5					
Median	4	5	3	**11**	3	2
Interquartile range	2–12	3–12	2–5	2–31	2–27	1–2
**Alkaline phosphatase—U/litre (n)**	114	47	44	18	3	2
Reference range	40–120					
Median	**152**	**163**	**157**	**143**	**152**	84
Interquartile range	107–243	121–287	87–240	94–231	70–158	79–89
**γ-Glutamyl transferase—U/litre (n)**	111	48	42	16	3	2
Reference range	0–35					
Median	**117**	**145**	**74**	**169**	**55**	**59**
Interquartile range	55–235	102–281	31–201	66–222	15–381	35–83
**Alanine transaminase—U/litre (n)**	132	58	47	22	3	2
**Reference range**	7–35					
**Median**	32.5	**40**	19	32.5	25	**115.5**
Interquartile range	19–56.5	30–78	13–35	23–61	18–33	33–198
**Aspartate transaminase—U/litre(n)**	102	45	40	12	3	2
Reference range	13–35					
Median	81.5	**153**	**35**	**77**	**39**	**245.5**
Interquartile range	35–173	91–272	23–61	37–115.5	33–44	157–334
**C-reactive protein (n)**	40	17	14	4	2	2
Reference range	<10					
Median	98	143	65	66	77	136.5
Interquartile range	42–175	67–210	32–116	52.5–172	3–151	136–137
**Full blood count**						
**Haemoglobin ×10−9/litre (n)**	160	69	61	22	4	3
Reference range	12.0–15.0					
Median	9.3	**8.1**	**10.8**	**8.3**	**9.2**	**11**
Interquartile range	7.25–11.2	6.6–9.9	9.1–12.4	6.8–10.2	8.75–11.25	7.4–11.3
**Red cell distribution width % (n)**	160	69	61	22	4	3
Reference range	11.6–14.0					
Median	16.2	**16.4**	**15.4**	**17.4**	**19.15**	**16.9**
Interquartile range	14.6–18.65	14.9–18.9	14.2–17.5	16.3–19.2	15.85–21.9	13.3–26.5
**White-cell count— ×10−9/litre (n)**	161	70	61	22	4	3
Reference range	4.00–10.00					
Median	5.32	4.96	6.07	**3.94**	5.39	**15.43**
Interquartile range	3.64–8.34	3.53–7.79	4.31–9.38	2.65–5.63	4.2–9.73	5.1–45.24
**Red-cell count— ×10−9/litre (n)**	160	69	61	22	4	3
Reference range	3.80–4.80					
Median	3.36	3	3.78	3.095	3.055	3.74
Interquartile range	2.63–3.915	2.28–3.54	3.1–4.18	2.63–3.77	2.62–3.805	2.58–14.2
**Neutrophil count— ×10−9/litre (n)**	94	40	39	12	2	1
Reference range	2.00–7.00					
Median	3.09	2.805	3.88	2.715	2.67	5.63
Interquartile range	2.18–5.8	1.525–4.305	2.4–10.21	1.49–5.055	2.24–3.1	5.63–5.63
**Lymphocyte count— ×10−9/litre (n)**	94	40	39	12	2	1
Reference range	1.00–3.00					
Median	0.73	**0.47**	1.1	**0.515**	1.14	**5.8**
Interquartile range	0.35–1.63	0.285–0.905	0.55–1.73	0.235–1.235	0.59–1.69	5.8–5.8
**Monocyte count— ×10−9/litre (n)**	94	40	39	12	2	1
Reference range	0.20–1.00					
Median	0.305	0.15	0.42	0.365	0.285	**1.9**
Interquartile range	0.14–0.6	0.09–0.405	0.2–0.74	0.22–0.56	0.2–0.37	1.9–1.9
**Eosinophil count— ×10−9/litre (n)**	92	39	39	12	2	
Reference range	0.00–0.40					
Median	0.075	0.09	0.08	0.035	0.085	
Interquartile range	0.02–0.195	0.02–0.2	0.03–0.26	0.01–0.27	0.03–0.14	
**Basophil count— ×10−9/litre (n)**	88	37	37	12	2	
Reference range	0.00–0.10					
Median	0.02	0.02	0.02	0.015	0.025	
Interquartile range	0.01–0.04	0.01–0.06	0.01–0.04	0.01–0.045	0.01–0.04	
**Platelet count— ×10−9/litre (n)**	159	68	61	22	4	3
Reference range	171–388					
Median	271	194.5	298	194.5	-	190
Interquartile range	148–386	122–351	211–402	61–419	-	173–484

### Blastomycosis

Two per cent (14/683) of the cases were diagnosed with blastomycosis. Five cases were diagnosed by culture plus phenotypic identification methods and another five were diagnosed by culture and phenotypic identification plus panfungal PCR/sequencing. A single case was identified by PCR/sequencing alone. Three cases were classified as probable cases because yeasts consistent with *Blastomyces* species were observed during histology examination of tissue samples. Ten of the 14 cases were male with a median age of 34 years (IQR, 31–50) (Table 1). The cases of blastomycosis were diagnosed in six of the nine provinces. Most cases were diagnosed in the Eastern Cape (6/14, 43%) and Western Cape provinces (4/14, 29%). One case each was diagnosed in Free State, KwaZulu-Natal, Gauteng and North West provinces (S1 Fig). Species identification was available for five cases, of which four were *B*. *percursus* based on panfungal PCR/sequencing, and one was *B*. *emzantsi* on panfungal PCR/sequencing. Skin tissue was the most common specimen type submitted for diagnosis of five cases. Four of the five patients had multiple skin abscesses and pulmonary involvement, while one patient had a brain lesion seen on a computed tomography scan of the head. HIV infection status was known for seven patients and five were HIV-seronegative. All patients with proven *B*. *percursus* and *B*. *emzantsi* infections were HIV-seronegative. No further clinical details were available for the other cases. Low serum albumin levels were reported in four patients and three patients had high serum levels of alkaline phosphatase, γ-glutamyl transferase and aspartate transaminase.

### Imported mycoses

Only four cases were diagnosed with *Talaromyces marneffei* infection. Three cases were reported in the Gauteng Province and one in KwaZulu-Natal Province. Two cases were diagnosed using culture plus phenotypic identification only, while the other two were diagnosed using culture and both phenotypic identification and PCR/sequencing. Two patients were male and the other two were female with a median age of 36 years (IQR, 29–39). Diagnosis was made from blood culture for three cases and pus from an abscess from one case. Of the four cases, three had a known HIV status and all were HIV-seropositive. Only one had a known CD4 count of 5 cells/μl. Two patients had an available travel history and confirmed recent travel to China. One was a South African woman who travelled to mainland China. She presented with hepatosplenomegaly and skin lesions on the face. The second patient was also a South African woman who worked in Hong Kong and her infection was diagnosed from pus from an abscess. High peripheral white cell counts were reported in three patients and elevated serum levels of alkaline phosphatase, γ-glutamyl transferase and aspartate transaminase were reported in two patients.

### Unspecified endemic mycoses

Most cases were not assigned to a causative fungal genus. Two hundred and seven cases were diagnosed as an unspecified endemic mycosis, possibly histoplasmosis or emergomycosis or blastomycosis because of the low or unknown specificity of the methods used. Seventy-seven per cent (160/207) were classified as probable cases and were diagnosed using the *Histoplasma* EIA (89/160, 56%), histopathology (68/160, 43%) and pan-dimorphic PCR assay (3/160, 2%) assay at NICD in 2020. None of these cases were culture-confirmed. Twenty-three per cent (47/207) were classified as possible cases. Among all patients with an unspecified endemic mycosis, 55% (114/207) had histology reports available and 79% (90/114) were reported as “deep fungal infections”. An HIV status for these patients was available for 60% (125/207) and of these, 98% (122/125) were HIV-seropositive.

### Laboratory survey on diagnosis of endemic mycoses at public hospitals

Of the 269 NHLS laboratories, only 21 (8%) microbiology and histopathology laboratories responded to the survey (S1 Text). From the 21 responses received, 15 indicated that they processed specimens from patients with a suspected endemic mycosis at least 1 to 5 times per annum and 3 reported diagnosing between 5 to 10 patients per year. All laboratories indicated regularly culturing *Sporothrix* and *Emergomyces*. Fifty per cent of the respondents indicated that they referred cases to other laboratories (such as the NICD) for confirmation. Tissue was the most common specimen type (75%) reported to be processed for diagnostic purposes. Specimens were reported to be processed in a class II biosafety cabinet by 81% of the respondents. Diagnostic methods used to identify endemic mycoses included culture (13, 62%) and histopathology (2, 10%).

## Discussion

Our analysis of pathology laboratory data resulted in the identification of 682 cases of an endemic mycosis, most of which were proven. Approximately a third of cases were categorized as an unspecified endemic mycosis as the causative pathogen could not be identified to genus level due to the limitations of the available diagnostic methods. Sporotrichosis was the most common endemic mycosis followed by emergomycosis, histoplasmosis, blastomycosis and rarely, talaromycosis, an imported mycosis. A majority was diagnosed using histopathology and the main specimen type submitted for diagnosis was skin tissue.

Our understanding of the pathogens causing endemic mycoses is that they are found in specific environmental niches and thus exposure and subsequent infection is generally associated with outdoor activities [32]. Histoplasmosis, blastomycosis and presumably emergomycosis follow inhalation of tiny airborne microconidia that convert to the yeast form in the lungs with dissemination to the skin and other organs or systems mainly in immunocompromised individuals [33]. Sporotrichosis is usually caused by traumatic inoculation of vegetative material containing the fungus into the skin [34]. Detailed data from skin test prevalence surveys, which serve as a proxy for environmental exposure, are rarely reported in South Africa. A report by Lurie *et al* in 1955 indicated 12% positive histoplasmin skin reactions among laboratory staff working at the South African Institute for Medical Research [35]. Oladele *et al* reported 12 histoplasmin skin test surveys conducted in Africa until 2017 with a positivity ranging from 0% to 35% [36, 37]. Mapping of laboratory-diagnosed cases does not generally indicate the source of infection, given the long incubation period between exposure and clinically-apparent disease and the fact that patients may access healthcare facilities distant from their places of work, residence or outdoor activities. In our study, we only report case numbers per province with no residential address or patient history to help identify potential environmental exposures. The provinces with most reported cases were Gauteng, Western Cape and KwaZulu-Natal; this could be related to their relatively larger populations but also to better health resources for case-finding and diagnosis. The distribution of cases was not uniform across the country with the coastal Western Cape Province reporting relatively more cases of emergomycosis; more cases of unspecified endemic mycoses and sporotrichosis in the high-altitude Gauteng Province; and relatively more cases of histoplasmosis in sub-tropical coastal province of KwaZulu-Natal. Based on very sparse underlying data, the burden of endemic mycoses in South Africa was roughly estimated at 100, 60, 40 and 10 cases per annum respectively for emergomycosis, histoplasmosis, sporotrichosis and blastomycosis [11]. As expected using laboratory-diagnosed cases, we report even fewer average cases per annum (15, 13, 17 and 1 for emergomycosis, histoplasmosis, sporotrichosis and blastomycosis, respectively). This could be attributed to cases being missed or misdiagnosed as TB, for example, because of the overlapping clinical features [38]. *T*. *marneffei* infections were only reported from patients who travelled outside of South Africa to known endemic regions.

Almost two thirds of the patients in our study were male, confirming previous reports that endemic mycoses are more common in males [39–41]. The number of laboratory-diagnosed cases increased from 2010 to a peak in 2015. This increase was mainly noted for emergomycosis and may have been related to the description of a novel fungus, *E*. *africanus* in South Africa in 2013 and related studies that improved case finding and diagnostic testing. This supports the link between better clinician awareness, improved diagnosis and case reporting. In 2020, a decrease in the number of detected cases could have been associated with COVID-19 pandemic travel restrictions that discouraged movement of people and thus many patients could not seek medical attention.

Oladele *et al* reported an association between histoplasmosis and HIV in Africa [24]. More than half of the patients in our study were HIV-seropositive. Emergomycosis and histoplasmosis were mainly diagnosed in people with advanced HIV disease, as previously reported [23,42–46]. This highlights the need for simple screening methods to facilitate an earlier diagnosis in advanced HIV, analogous to the approach used for HIV-associated cryptococcosis. Furthermore, half of the culture-confirmed sporotrichosis cases in our study were also HIV-seropositive. HIV-infection worsens sporotrichosis, resulting in more severe disseminated cases, higher need for hospitalization and risk of death [47]. However, some of these cases of sporotrichosis were only proven by culture plus phenotypic methods; inexperienced laboratory workers might misidentify *Sporothrix* cultures as *Emergomyces* or vice versa due to the morphological similarities between these fungi. Other than the association of histoplasmosis with HIV, no other risk factors were discussed in our study as it was beyond our scope. We recommend that more studies be done in order to understand all the risk factors associated with endemic mycoses in South Africa or the whole continent.

The distribution of endemic mycoses globally has been described recently leading to redefined geographical boundaries of these diseases [20]. Reports on endemic mycoses were previously mainly from the Americas, Asia and some parts of Africa [9,18,24,45]. In Africa, few countries have surveillance systems for endemic mycoses [48,49]. A few outbreaks have been reported on sporotrichosis and histoplasmosis among mine workers and laboratory workers respectively [22,35]. Outbreaks of acute pulmonary histoplasmosis in South Africa have occurred mostly in individuals who had recently explored caves [50,51]. Thermally-dimorphic fungal infections are associated with significant mortality and morbidity in individuals with compromised immune systems, with up to 50% mortality being reported in patients with AIDS [11,40,43]. Of the 50 patients with available outcome data in our study, 62% died. Most of these outcome data were for 39 patients with HIV-associated emergomycosis, of whom 55% died. Similarly high mortality (50% case fatality among 52 patients) was reported in a previous case series from South Africa including many of the same cases [43]. Schwartz *et al* reported a case series of 25 patients with endemic mycoses in the Western Cape Province, of whom 24% died; this lower overall mortality was attributed to early diagnosis [40]. This mortality associated with endemic mycoses in South Africa is much higher than the reported rate in the United States of 5% and 7% in children and adults respectively [52] and this may be related to HIV as a major underlying condition, late diagnosis and lack of appropriate treatment.

The diagnosis of endemic mycoses can be challenging and requires a multifaceted approach [53–56]. In South Africa, endemic mycoses are diagnosed at most pathology laboratories by traditional methods such as culture with morphological identification or histopathology, the gold standard methods [12]. Difficult-to-identify fungi are submitted to the NICD for confirmation by culture and molecular methods. A direct pan-dimorphic RT-qPCR or/and *Histoplasma* antigen EIA are performed on request at NICD. The histology laboratories employ stains such as periodic acid–Schiff (PAS), calcofluor white, Giemsa and Grocott’s methenamine silver for identification of yeasts in tissue specimens. In this study, most cases were diagnosed using histology alone and resulted in most cases being classified as an unspecified endemic mycosis. Histology alone generally cannot clearly differentiate among thermally-dimorphic fungi due to morphological similarities between *Histoplasma*, *Emergomyces*, *Blastomyces* and *Sporothrix* [57–58]. The proven cases in this study were diagnosed using culture with phenotypic identification alone or with molecular methods in some cases. Identification of fungi requires expertise and biosafety level 3 facilities for culturing of pathogens such *Blastomyces*, *Emergomyces* and *Histoplasma* are not always available. Both public and private laboratories recorded proven cases after culture with identification of fungi often only to a genus level; considering the similarities in morphology among dimorphic fungi, some of the confirmed cases in our study might not be correctly identified as recorded. Diagnostic laboratories reported thermally-dimorphic fungi cultured from nail and hair specimens for a few cases which is highly unlikely. This further highlights the problem of misidentification and lack of expertise in correctly identifying dimorphic fungi. There is need for constant training in laboratories to ensure correct identification. Our survey indicated that most public-sector laboratories relied on culture methods and/or histology for diagnosis of endemic mycoses and most depended on referring cases to the NICD reference laboratory for confirmation. However, from 2012 to 2021, only 167 (24%) cases were referred to the NICD. Forty per cent of isolates were incorrectly identified or listed as a thermally-dimorphic fungus with no genus/species identification. This further emphasizes the need for training. The NICD has one of very few mycology reference laboratories on the African continent, highlighting a general lack of surveillance and reference diagnostic capacity that may contribute to many cases being missed or misdiagnosed.

In our survey, rapid molecular and serological methods were not readily available at diagnostic laboratories. However, such assays are imperfect. Our pan-dimorphic PCR assay cannot differentiate among cases of infection caused by *Histoplasma*, *Blastomyces* and *Emergomyces* [29]. The close phylogenetic relationship of these different thermally-dimorphic fungi means that pathogen-specific assays are difficult to develop. Delays in diagnosis have consequences that may be fatal, can lead to unnecessary antimicrobial use which can result in resistance or can result in longer hospital admissions. Up to 16% patients in our study had multiple skin lesions. Although non-specific, this should trigger suspicion of an endemic mycosis in the differential diagnosis and prompt ordering of appropriate laboratory tests. The mycoses endemic to southern Africa are all treated in the same way with initial amphotericin B in severe cases, followed by itraconazole; moderate or mild cases are treated with itraconazole only [16,30,46]_._ One of the limitations of our study was that we did not have access to treatments records for the patients that would have given more clarity on how the diseases were treated.

A major strength of this study was its national coverage and use of diagnostic pathology laboratory data from both public and private sectors and a national reference laboratory. Some of the limitations included the search strategy used in the laboratory information systems. For example, histology results were searched using SNOMED clinical terms or the names of the thermally dimorphic fungi or diseases and some cases could have been missed. Our mapping of laboratory-diagnosed cases by province cannot be used to infer areas of higher or lower endemnicity. We also collected very limited demographic and clinical data in this retrospective laboratory surveillance. Characteristics such as ethnic group, underlying medical condition and immunosuppressing medicines were not consistently available. Travel history and data on occupational and recreational pursuits were also generally not available. Outcomes were also available for only 7% of cases. The results from biochemical and liver function tests for patients closer to the time of diagnosis was also missing for most patients and this was a limitation in terms of conclusions that could be reached using this data. We did not store FFPE tissue or cultured isolates from proven cases identified only at diagnostic laboratories for further confirmatory testing at the NICD; if we had, this would have confirmed the diagnosis and furthermore identified the gaps in diagnostic capabilities.

## Conclusion

This is a first attempt at determining the national epidemiology of endemic mycoses in South Africa. Although relatively few laboratory-diagnosed cases were identified, there is a large immunosuppressed population at risk of severe disease and ours is probably a minimum estimate. There is a clear need for careful environmental mapping of the pathogen reservoirs and active human and veterinary surveillance of cases in South Africa in order to understand the true burden of these diseases. Rapid and accurate serology and molecular diagnostic tests, which are appropriate for resource limited settings, will facilitate such surveillance. Mandatory reporting of endemic mycoses would also improve surveillance efforts but would also increase the reporting burden on diagnostic laboratories. More funding needs to be invested into neglected endemic mycoses to address the knowledge gaps in this field. Programs are also needed to improve laboratory diagnosis of endemic mycoses in Africa. Awareness of these diseases should also be increased in the general population and among healthcare workers.

## Supporting information

S1 Fig Distribution of endemic mycoses in South Africa by province from 2010 to 2020. (TIF)

S1 Text Endemic Mycoses in South Africa survey. (DOCX)
