# Supplementary figures and images for: Endemic mycoses in South Africa, 2010–2020: A decade-long description of laboratory-diagnosed cases and prospects for the future
Source: PLoS Negl Trop Dis. 2022 Sep 28;16(9):e0010737. doi: 10.1371/journal.pntd.0010737 (PMC9518919; doi:10.1371/journal.pntd.0010737)

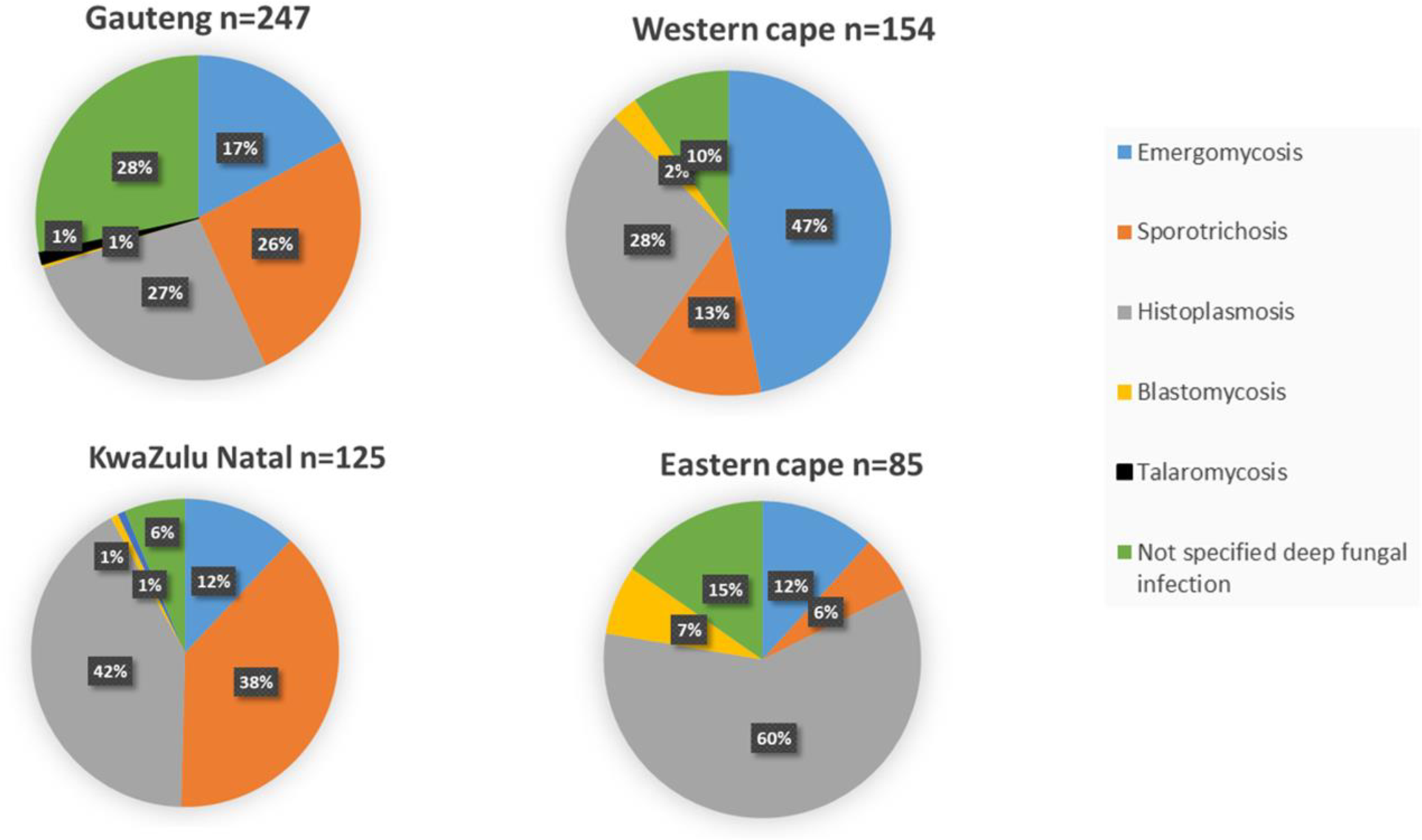

Supplement: S1 Fig — (TIF) [file pntd.0010737.s001.tif]
