# Supplementary material for: Endemic mycoses in South Africa, 2010–2020: A decade-long description of laboratory-diagnosed cases and prospects for the future
Source: PLoS Negl Trop Dis. 2022 Sep 28;16(9):e0010737. doi: 10.1371/journal.pntd.0010737 (PMC9518919; doi:10.1371/journal.pntd.0010737)
Supplement: S1 Text — (DOCX) [file pntd.0010737.s002.docx]

| **S1 Text.** [**Endemic Mycoses in South Africa survey**](https://docs.google.com/forms/d/e/1FAIpQLSf3D46oUYlWmWhL4eYZ-AKLGPzCvYrBF4LHHe4eVQjDQhjbdw/viewform?vc=0&c=0&w=1&flr=0&usp=mail_form_link) |
| --- |
|  |
| I would like to invite you to kindly participate in a survey as part of our evaluation and surveillance on infections caused by thermally dimorphic fungi. A brief background on thermally-dimorphic fungi is below:  Thermally dimorphic fungi are a unique group of fungi within the Ascomycota phylum that respond to shifts in temperature by converting between hyphae (22–25°C) and yeast (37°C). This morphologic switch, known as the phase transition, defines the biology and lifestyle of these fungi. Thermally-dimorphic fungi including *Histoplasma capsulatum*, *Emergomyces* species, *Coccidioides immitis*, *Blastomyces dermatitidis*, *Paracoccidioides* species, *Sporothrix* species and *Talaromyces marneffei* cause life-threatening diseases, particularly in immunosuppressed hosts. Most thermally-dimorphic fungal infections are acquired through the respiratory route, with the exception of *S. schenckii* complex, which can also be acquired through traumatic inoculation. Transmission does not occur from person-to-person. Infection usually occurs following inhalation of soil contaminated with conidia and these conidia lodge in the lung or may disseminate to other organs of the body.   In emergencies please contact us at 011 555 0325 or [ruthm@nicd.ac.za](mailto:ruthm@nicd.ac.za) |
|  |
| **Name of laboratory ***  **How often do you diagnose infections caused by thermally dimorphic fungi per year? * ***   - - ( ) 0   - ( ) 1-5   - ( ) 5-10   - ( ) 10-15   - ( ) 15-20   - ( ) Other:   **Can you choose the dimorphic infections you diagnosed in your lab in the past year. * ***   - - ( ) Histoplasmosis   - ( ) Blastomycosis   - ( ) Sporotrichosis   - ( ) Emergomycosis   - ( ) Talaromycosis   - ( ) none   - ( ) Other:   **Do you process the requests or refer them to other laboratories for identification? * ***   - - ( ) Yes   - ( ) No   - ( ) If yes please specify below   - ( ) Other:   **Which type of specimen is normally sent to the laboratory for diagnosis of thermally-dimorphic fungal? * ***   - - ( ) bone marrow   - ( ) skin   - ( ) blood   - ( ) tissue biopsy   - ( ) cerebrospinal fluid   - ( ) pus   - ( ) Other:   **Where do you process your specimens? ***   - - ( ) On the bench   - ( ) Under the Biosafety cabinet   - ( ) Other:   **Which methods do you use for diagnosis of thermally-dimorphic fungi? ***   - - ( ) Cytopathology   - ( ) Histopathology   - ( ) Culture   - ( ) panfungal ITS PCR   - ( ) real-time PCR   - ( ) enzyme immune assay   - ( ) Other:   **How long do you normally keep the culture plates when suspecting a thermally dimorphic fungus in your laboratory before discarding these, if there is no fungal growth? ***   - - ( ) Less than 5 days   - ( ) 5 - 10 days   - ( ) 3 weeks   - ( ) 1 month   - ( ) Other:   **What are your challenges when identifying thermally dimorphic pathogens in your laboratory? ***   - - ( ) lack of skilled person to ID these pathogens   - ( ) not knowledgeable about these pathogens   - ( ) Other:   **How do you think this challenges can be overcome to improve diagnosis of thermally-dimorphic fungi? Provide training on identification of these pathogens ***  **How do you think the NICD can assist with such diagnostics? ***  **Are you happy with the service being provided by the Mycology Reference Laboratory at the NICD? ***   - - ( ) Yes   - ( ) No   - ( ) Maybe   **Can you please rate our service from 1 to 5. ***   \|  \| 1 \| 2 \| 3 \| 4 \| 5 \|  \| \| --- \| --- \| --- \| --- \| --- \| --- \| --- \| \| poor \| ( ) \| ( ) \| ( ) \| ( ) \| ( ) \| excellent \|   **Any other comments ***  **Name of laboratory ***  **How often do you diagnose infections caused by thermally dimorphic fungi per year? * ***   - - ( ) 0   - ( ) 1-5   - ( ) 5-10   - ( ) 10-15   - ( ) 15-20   - ( ) Other:   **Can you choose the dimorphic infections you diagnosed in your lab in the past year. * ***   - - ( ) Histoplasmosis   - ( ) Blastomycosis   - ( ) Sporotrichosis   - ( ) Emergomycosis   - ( ) Talaromycosis   - ( ) none   - ( ) Other:   **Do you process the requests or refer them to other laboratories for identification? * ***   - - ( ) Yes   - ( ) No   - ( ) If yes please specify below   - ( ) Other:   **Which type of specimen is normally sent to the laboratory for diagnosis of thermally-dimorphic fungal? * ***   - - ( ) bone marrow   - ( ) skin   - ( ) blood   - ( ) tissue biopsy   - ( ) cerebrospinal fluid   - ( ) pus   - ( ) Other:   **Where do you process your specimens? ***   - - ( ) On the bench   - ( ) Under the Biosafety cabinet   - ( ) Other:   **Which methods do you use for diagnosis of thermally-dimorphic fungi? ***   - - ( ) Cytopathology   - ( ) Histopathology   - ( ) Culture   - ( ) panfungal ITS PCR   - ( ) real-time PCR   - ( ) enzyme immune assay   - ( ) Other:   **How long do you normally keep the culture plates when suspecting a thermally dimorphic fungus in your laboratory before discarding these, if there is no fungal growth? ***   - - ( ) Less than 5 days   - ( ) 5 - 10 days   - ( ) 3 weeks   - ( ) 1 month   - ( ) Other:   **What are your challenges when identifying thermally dimorphic pathogens in your laboratory? ***   - - ( ) lack of skilled person to ID these pathogens   - ( ) not knowledgeable about these pathogens   - ( ) Other:   **How do you think this challenges can be overcome to improve diagnosis of thermally-dimorphic fungi? Provide training on identification of these pathogens ***  **How do you think the NICD can assist with such diagnostics? ***  **Are you happy with the service being provided by the Mycology Reference Laboratory at the NICD? ***   - - ( ) Yes   - ( ) No   - ( ) Maybe   **Can you please rate our service from 1 to 5. ***   \|  \| 1 \| 2 \| 3 \| 4 \| 5 \|  \| \| --- \| --- \| --- \| --- \| --- \| --- \| --- \| \| poor \| ( ) \| ( ) \| ( ) \| ( ) \| ( ) \| excellent \|   **Any other comments *** |
|  |
|  |
